# Prevalence and factors associated with musculoskeletal disorders among dental practitioners in Rwanda

**DOI:** 10.6026/973206300222756

**Published:** 2026-05-31

**Authors:** Dona Soman, Danilo Milanes Zambrano, Ramnath Elangovan, Varun Chopra, Bienvenu Emile, Ishimwe Nicole, Twizerimana Ange

**Affiliations:** 1 Department of Prosthetic and Restorative Dentistry, School of Dentistry, University of Rwanda, Kigali, Rwanda;; 2 Department of Orthodontics and Pediatric Dentistry, School of Dentistry, University of Rwanda, Kigali, Rwanda, East Africa;; 3 Department of Periodontics and Community Dentistry, School of Dentistry, University of Rwanda, Kigali, Rwanda, East Africa;; 4 Department of Oral and Maxillofacial Surgery, Medicine and Pathology, School of Dentistry, University of Rwanda, Kigali, Rwanda,East Africa;; 5 Department of Dental Surgery, School of Dentistry, University of Rwanda, Kigali, Rwanda, East Africa;

**Keywords:** Musculoskeletal disorders (MSDs), dental practitioners, ergonomics, occupational hazards, work related musculoskeletal disorders

## Abstract

Musculoskeletal disorders among dental professionals remain highly prevalent due to prolonged non-ergonomic postures, repetitive movements and inadequate work practices. Therefore, it is of interest to assess the prevalence and associated factors of musculoskeletal disorders among 268 dental practitioners in Rwanda using stratified random sampling and questionnaire-based data analyzed with SPSS version 27. The overall prevalence of musculoskeletal disorders was 84%, with the neck (64.0%), lower back (62.7%) and wrists or hands (42.2%) being the most affected regions. Significant associations were observed with repetitive movements, insufficient breaks between procedures and increased working days per week (p < 0.05). Thus, we show the need for ergonomic interventions and preventive strategies to reduce occupational health risks among dental practitioners.

## Background:

Dentistry is a hard field that requires a high level of focus and accuracy. The capacity to sustain occupational postures for extended periods of time, psychomotor abilities, depth perception, hearing, manual dexterity and visual acuity are all necessary for dentists [1]. A disorder of muscles, tendons, peripheral nerves, or vascular system not directly resulting from an acute or instantaneous event (e.g., slips or falls) is how the World Health Organization (WHO) defines MSD [2]. Poor posture and improper positioning are the primary factors contributing to musculoskeletal (MS) pain in dentists. The likelihood of developing musculoskeletal disorders (MSDs) in dentistry can be reduced by following ergonomic guidelines. A significant link exists between physical ergonomics and the occurrence of MSDs among dental health professionals [3]. In many studies, only a limited number of these factors have been considered at the same time. This complicates the understanding of how specific factors influence outcomes, as most research has not adequately controlled for overlapping factors. In Greece, there are only a handful of studies examining the simultaneous presence of various musculoskeletal issues in different occupational groups and how these issues are related to one another [4]. Several studies have indicated that musculoskeletal diseases and pain significantly lead to lower productivity, diminished work quality and reduced job satisfaction, increased occupational accidents, sick leave and early retirement from the profession. Additionally, these conditions can incur substantial medical treatment costs. For example, in Germany, the medical expenses related to musculoskeletal diseases and pain reached 34.2 billion euros in 2015, accounting for 10.1% of total healthcare spending [5]. However, MSDs also appear to be more common in females than in males. Historically, the dental field has been largely male dominated. Today, the gender ratio among dentists is relatively balanced, with 45.6% being female and 54.4% male. However, with the increasing number of female dental students, demographic shifts are starting to occur. In Germany, the number of female dental students is nearly double that of their male counterparts and this trend is on the rise. As a result, it is essential to focus on the specific needs of female dentists [6].

The issue of MSDs is critical all over the world. Dental professionals worldwide report varying rates of musculoskeletal disorders (MSDs). A study by Bret and Gorse found that over 60% and 35-55% of dentists experienced MSDs in the lower back and upper extremities, respectively. The highest prevalence of MSDs was found in the neck (82%) and lower back (64%) among dentists in Malaysia, with female dentists showing a greater susceptibility to these disorders [7]. Dentists are obliged daily to maintain prolonged static positions and engage in repetitive movements of the neck, arms, shoulders and hands, whether sitting or standing. This can lead to discomfort in different areas of the body. If these recurring discomforts are overlooked, they can result in long-term musculoskeletal and psychological issues, ultimately affecting performance and productivity [8]. Musculoskeletal disorders (MSDs) can be triggered by non-ergonomic working positions, such as prolonged head rotation, neck bending and shoulder and upper arm abduction, along with repetitive and sustained high-intensity static loads on muscles and joints. Additionally, psychosocial risk factors and individual traits can heighten the likelihood of developing MSDs. Common symptoms of occupational MSDs include neck pain and motor dysfunction, which are major contributors to disability and impairment, affecting both the work and personal lives of dentists [9]. Therefore, it is of interest to assess the prevalence and factors associated with musculoskeletal disorders (MSDs) among dental practitioners working in Rwanda.

## Materials and Methods:

This chapter discusses in detail the following sections: Study area, study design, study population, study sample size, selection criteria, data collection, data analysis and ethical consideration.

## Study area:

study design, study population, study sample size, selection criteria, data collection, data analysis and ethical consideration.

## Study design:

Research design is a cross-sectional study. In this study, quantitative data collected using self-structured questionnaire containing factors of MSDs related questions that respond to the study objectives.

## Study population:

The targeted population was licensed and practicing dental practitioners working in facilities providing dental care in various area of Rwanda. There are 324 licensed dental therapists practicing in Rwanda as reported by one of the leaders of Rwanda dental association and 116 dental surgeons (obtained from RMDC website).

## Study sampling:

This study employed Stratified Random Sampling to ensure balanced representation among dental practitioners in Rwanda, focusing on professional roles. The population was divided into two strata: dental therapists and dental surgeons.

The total sample size is set at 268, calculated using Yamane's formula, so the samples size is calculated in each stratum and allocated as follows:

[1] 178 dental therapists (from a total of 322)

[2] 90 dental surgeons (from a total of 116)

A single Google Form was distributed in different WhatsApp groups for dental therapists and dental surgeons. Participants included based on their professional role, with the first 178 dental therapists and the first 90 dental surgeons who completed the survey meeting the required quotas.

## Sample size:

In this study, the sample size for dental practitioners in Rwanda was calculated using Yamane's formula, which is appropriate when the population size is known. The total population of dental practitioners was 440, consisting of 324 dental therapists and 116 dental surgeons. The sample size (n) was determined using a 5% margin of error (e = 0.05), which corresponds to a 95% confidence level. By substituting the values into the formula, the required sample size was calculated to be approximately 268 participants. The sample was proportionally allocated according to professional category, resulting in 178 dental therapists and 90 dental surgeons. This approach ensured sufficient and representative sample size for reliable statistical analysis.

amane's formula is expressed as:

n=N/1+N (e2)

Where:

n = required sample size

N = total population size

e = margin of error (0.05)

## Inclusion criteria:

[1] Dental practitioners actively working in dental clinics and health facilities in Rwanda.

[2] Dental practitioners who voluntarily agree to participate in the study.

## Exclusion criteria:

[1]Retired or inactive dental practitioners who are no longer involved in clinical dental practice.

[2]Administrative staff in dental clinics that do not provide dental care services.

## Data collection:

Data collected using a self-developed questionnaire consisting of demographic information, exposure and factors associated with MSDs among dental practitioners. The questionnaire was designed based on literature related to our topic of study. It was presented as Google form to participants and shared in each WhatsApp groups of all registered dental therapists and dental surgeons

## Data analysis:

Data was analysed by descriptive statistics using IBM SPSS statistics software version 27, univariate analysis to calculate the frequencies, percentages. Bivariate analysis was used to determine the association between variables. Ethical considerations Ethical clearance sought from the CMHS Institutional Review Board (IRB). The study team informed participants that their involvement is voluntary and they are free to withdraw at any time. Privacy, confidentiality and anonymity were ensured by not pressuring participants to respond, securely storing all information, using it solely for research purposes.

## Results:

**Figure 1:**
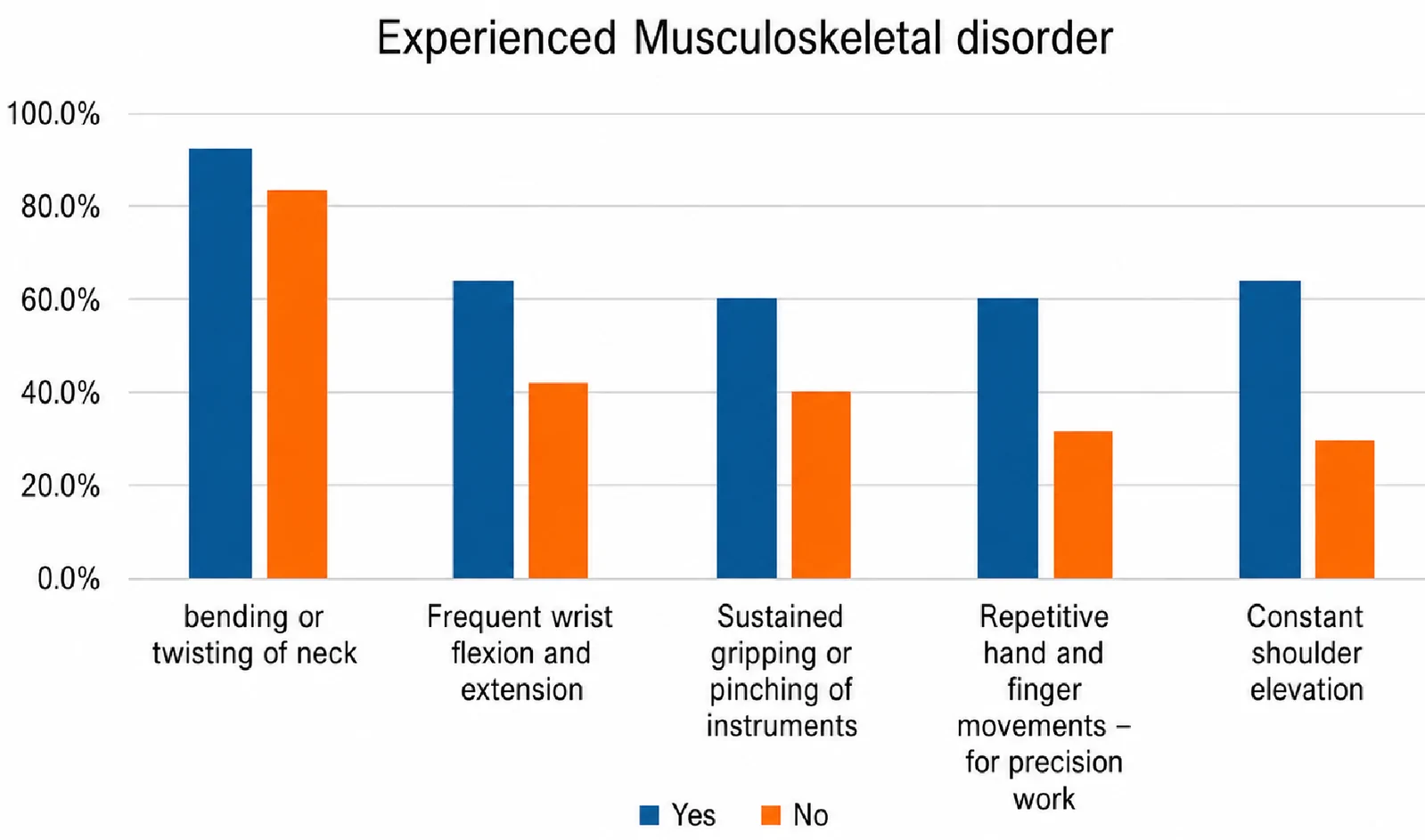
Association of MSDs and repetitive movement type involvement

This chapter presents the findings of the study. It is divided into parts including demographic characteristics, Prevalence of MSDs, association demographic and work-related factors with MSDs Prevalence. Demographic characteristics a total of 268 participants were included in the study. Most respondents were aged between 30-39 years (51.5%), followed by those aged 20-29 years (27.6%). A smaller proportion fell within the age ranges of 40-49 years (17.2%) and 50 years and above (3.7%). The study population was predominantly male (73.5%). Regarding professional experience, most participants had 6-10 years of practice (38.8%), followed by 0-5 years (31.7%), 11-15 years (22.4%) and 16 years or more (7.1%). In terms of professional category, dental therapists made up the largest group (66.4%), while dental surgeons and specialists accounted for 27.6% and 6.0%, respectively. Geographically, over half of the respondents were based in Kigali City (55.6%), with the remainder distributed across the four provinces of Rwanda. Most participants worked in private clinics (42.5%), followed by district hospitals (28.7%), referral hospitals (12.7%) and community health centers (3.4%). A small portion reported working across multiple facility types (12.7%). Concerning religion, the majority identified as Christian (65.7%), with Adventists (18.3%), Muslims (11.2%) and other faiths (4.9%) also represented Table 1. The findings revealed a high prevalence of musculoskeletal disorders (MSDs) among the study participants, with 84.0% (n = 225) reporting having experienced at least one form of MSD, while only 16.0% (n = 43) reported no such experience. The most affected anatomical regions were the neck (64.0%), lower back (62.7%) and wrists or hands (42.2%). Other reported areas included the upper back (31.6%), shoulders (51.6%), hips (8.4%), elbows (7.6%), feet or ankles (8.9%) and knees (5.3%), indicating that a substantial proportion of respondents experienced discomfort in multiple body parts. In terms of frequency, most participants (66.7%) experienced musculoskeletal pain several times a week, while 16.0% reported weekly pain, 12.4% monthly and 4.9% experienced pain daily. Regarding pain severity, over half of the respondents (51.6%) categorized their pain as moderate, 43.1% as mild and only 5.3% as severe. These results suggest that MSDs are a widespread occupational health concern within this population, with potential implications for workforce well-being, clinical efficiency and long-term physical health. The high frequency and moderate intensity of reported symptoms highlight the urgent need for preventive strategies and ergonomic interventions within the practice environment Table 2, Table 3 illustrates that there were no significant relationships between musculoskeletal disorders (MSDs) and the tested demographic factors in the study. Although the highest prevalence of MSDs was observed among workers in the 40-49-year age group (95.7%), followed by 30-39 years (82.6%) and 20-29 years (79.7%), the correlation between age and prevalence of MSDs was not statistically significant (p = 0.114). Gender did not also have a significant difference, as MSD prevalence was 84.3% in males and 83.1% in females (p = 0.819). Years of practice likewise did not have a significant effect on the occurrence of MSD (p = 0.240), even though a slightly greater prevalence was observed for those with 6-10 years of experience (89.4%). Regarding practice type, all the dental specialists reported MSDs (100%), compared to 83.7% of dental therapists and 81.1% of dental surgeons, but the difference was not significant (p = 0.172). While MSD prevalence was higher in Kigali City (86.6%) than outside Kigali (73.6%), this geographical distinction was not statistically significant (p = 0.340). Health facility type approached significance (p = 0.057), with referral hospitals recording the highest prevalence (97.1%) and community health centers recording the lowest (66.7%). Religion was not significantly linked to MSD prevalence (p = 0.224), despite variation in prevalence across different religious affiliations Table 3 The examination of work-related factors Table 4 has uncovered several statistically significant correlations with the occurrence of musculoskeletal disorders (MSDs) among dental professionals. Specifically, the number of working days each week was significantly correlated with MSDs (p = 0.009), with a greater prevalence noted among individuals working 4 to 7 days weekly. Additionally, the presence of arm support on the operator's chair demonstrated a significant connection with MSDs (p = 0.010). Practitioners utilizing chairs lacking armrests reported a higher incidence of MSDs compared to those with arm support. The frequency of breaks taken between dental procedures was also found to be highly significant (p < 0.001). Participants who did not take breaks experienced elevated rates of MSDs, while those who took breaks every 2 to 4 hours reported fewer symptoms. Moreover, engagement in repetitive movements was significantly linked to MSDs (p < 0.001). Practitioners engaged in tasks that required constant bending, wrist movements, or gripping instruments reported a higher prevalence of MSDs, emphasizing the impact of repetitive strain on the onset of these conditions. Despite a slightly higher proportion of MSD experience among participants without reported pre-existing conditions, the association between pre-existing conditions and MSD occurrence was not statistically significant (p= 0.224). Other elements such as daily working hours, the number of patients treated per day, the performance and duration of hand scaling, chair adjustability, the presence of back support and pre-existing conditions did not exhibit statistically significant associations (p > 0.05). The bar chart 1 illustrates the correlation between certain repetitive movements and the prevalence of musculoskeletal disorders (MSDs) among dental professionals. The highest rate of MSDs was noted in individuals who often performed neck bending or twisting (93.5%), followed by those with constant shoulder elevation (62.5%), frequent wrist flexion and extension (62.0%), repetitive hand and finger movements for precision tasks (58.5%) and prolonged gripping or pinching of instruments (57.5%). These results indicate that repetitive upper-body movements, particularly those involving the neck and shoulders are significantly linked to the development of MSDs in dental practitioners, highlighting the necessity for ergonomic solutions in everyday clinical practice Figure 1.

## Discussion:

Prevalence of musculoskeletal disorders among dental practitioners in Rwanda. The current study revealed a high prevalence of musculoskeletal disorders (MSDs) among dental practitioners in Rwanda, with 84.0% of participants reporting symptoms, which aligns closely with the 73% to over 90% prevalence rates reported in a study conducted by Al-Huthaifi et al. 2023 [10]. The most affected body regions in our study were the neck (64.0%), lower back (62.7%) and wrists/hands (42.2%). This distribution is consistent with previous findings that highlight the neck and lower back as the primary sites of discomfort due to sustained awkward postures and repetitive strain during dental procedures; For example, the prevalence of neck pain in our study mirrors earlier reports where the neck region scored the highest discomfort levels due to overuse and inflammation from prolonged static positioning. Similarly, the lower back discomfort rates in our findings (62.7%) are comparable to the 30-33% range reported in other studies, confirming that prolonged spinal bending and torso extension are key contributing factors [5]. The moderate pain severity and frequency of symptoms experienced several times a week by most participants also reflects patterns seen in other research, where chronic complaints and fatigue were common among dentists. Despite the high burden of MSDs, awareness and application of ergonomic principles remain limited in our study population, which concurs with previous reports emphasizing inadequate ergonomic knowledge and insufficient rest breaks as important risk factors for MSD development [11]. Taken together, these comparisons reinforce the need for targeted ergonomic interventions and education programs to reduce MSD prevalence and improve occupational health among dental professionals in Rwanda. Association of demographics and MSDS development in our study, we did not find any significant links between demographic factors and the onset of musculoskeletal disorders (MSDs) among dental professionals in Rwanda. While certain studies from Southeast Turkey, Australia and various systematic reviews have indicated a positive correlation between advancing age and the incidence of MSDs [12, 13], as well as a negative correlation with years of professional practice and clinical experience [14]. Our results did not demonstrate any statistically significant relationship between age or years of experience and the prevalence of MSDs. Moreover, although Bozkurt et al. identified a significant link between gender and MSDs suggesting that females are at a higher risk. Our study revealed comparable rates of MSDs among both male and female practitioners, indicating no substantial relationship between gender and the emergence of musculoskeletal disorders within our study group. Association of work-related factors and MSDs the analysis of work-related factors revealed that certain occupational practices and ergonomic conditions were significantly associated with the occurrence of musculoskeletal disorders among dental practitioners. Notably, engaging in repetitive movements, working more days per week, taking fewer or no breaks during procedures and the lack of arm support on the operator's chair were all significantly associated with higher reports of MSDs. These findings underscore the critical impact of cumulative physical stress and inadequate ergonomic support in clinical practice. The examination of work-related factors has uncovered several statistically significant correlations with the occurrence of musculoskeletal disorders (MSDs) among dental professionals. Specifically, the frequency of breaks taken between dental procedures showed a statistically significant association with the occurrence of musculoskeletal disorders (MSDs) (p < 0.001). Participants who reported not taking breaks experienced notably higher rates of MSDs, whereas those who paused every 2 to 4 hours demonstrated a lower prevalence of symptoms. This finding highlights the critical role of scheduled rest periods in minimizing musculoskeletal strain and fatigue. These results are consistent with findings by Lietz *et al.* who reported that the lack of regular breaks between dental tasks significantly increased the risk of musculoskeletal pain, underscoring how work schedules can influence the development of such disorders [5]. A statistically significant correlation was also identified between the number of working days each week and the occurrence of musculoskeletal disorders (MSDs) (p = 0.009), with elevated rates noted among individuals working four to seven days. This implies that a lack of adequate rest between workdays could play a role in the onset of musculoskeletal disorders. Research shows that consistently working in same posture without sufficient recovery time heightens physical strains [8]. Consequently, the inclusion of regular days off may be essential in reducing the risk of MSDs among dental practitioners. Similarly, statistically significant correlation was found between musculoskeletal disorders (MSDs) and the lack of arm support on the operator's chair (p = 0.010). This result indicates that inadequate ergonomic design, particularly the absence of armrests, may lead to heightened physical strain during dental procedures. Adequate ergonomic seating, which includes the provision of arm support, is crucial for improving practitioner comfort and reducing the likelihood of developing MSDs. Lietz *et al.* noted that ergonomic interventions, such as the use of well-designed chairs, considerably alleviated musculoskeletal symptoms in individuals involved in extended seated work activities [5].

Moreover, it was discovered that engagement in repetitive movements has a strong and statistically significant correlation with musculoskeletal disorders (MSDs) (p < 0.001). Centrally, even though the frequence of engagement was high among those who frequently engaged (50.0%) there was no significance between MSDs development and frequence of repetitive movement engagement. Dental professionals who regularly performed actions such as bending or twisting their necks, repeatedly flexing and extending their wrists, maintaining a grip or pinch on instruments and keeping their shoulders elevated for extended periods reported a higher incidence of MSDs. These results underscore the vital importance of repetitive strain in the onset of musculoskeletal problems. In a similar vein, Shah *et al.* noted that dentist who habitually maintains awkward postures whether seated or standing experience heightened stress on body areas, especially due to excessive neck bending and unnatural twisting of the lumbar region, which consequently increases the risk of MSDs [8]. In support of this, Lietz et al. also indicated that holding awkward postures for more than 40 minutes significantly exacerbates musculoskeletal strain [5]. On the other hand, In our study, various occupational factors including daily working hours, the number of patients treated per day, the frequency and duration of manual hand scaling, chair adjustability, the presence of back support and pre-existing health conditions were not found to have statistically significant associations with the development of musculoskeletal disorders (MSDs) (p > 0.05). However, contrasting findings were reported by Lietz *et al.* who observed significant relationships between MSDs and factors such as the number of patients treated daily and, also the performance and frequency of hand scaling procedures [5]. Shah *et al.* study also indicated that working more than eight hours per day significantly increased the risk of musculoskeletal disorders development [8]. However, the adjustability of both the operator's and the patient's chairs found to influence the MSDs development.

## Conclusion:

Musculoskeletal disorders (MSDs) are a major occupational health concern among dental practitioners due to prolonged static postures and the physically demanding nature of dental procedures. In this study, approximately 84% of dental practitioners in Rwanda reported experiencing MSDs, with the neck, lower back and shoulders being the most affected areas. The high prevalence was associated with factors such as long working hours, repetitive movements, poor working posture and inadequate ergonomic practices, highlighting the need for improved ergonomic awareness and preventive strategies.

## Figures and Tables

**Table 1:** Demographic characteristics

**Variables**	**Categories**	**Frequency**	**Percentages**
		**(n)**	**(%)**
Age In Years	20-29	74	27.6
	30-39	138	51.5
	40-49	46	17.2
	≥50	10	3.7
Gender	Male	197	73.5
	Female		
Years Of Practice	0-5	85	31.7
	10-Jun	104	38.8
	15-Nov	60	22.4
	16 And above	19	7.1
Type Of Practice	Dental surgeon	74	27.6
	Dental therapist	178	66.4
	specialist	16	6
Geographical Area	Kigali city	149	55.6
	out of Kigali	119	44.4
Facility Type	District hospital	77	28.7
	Referral hospital	34	12.7
	Private clinic	114	42.5
	Community health centers	9	3.4
	Multiple facility types	34	12.7
Religion	Christian	176	65.7
	Adventist	49	18.3
	Muslim	30	11.2
	Others	13	4.9

**Table 2:** MSDs prevalence

**Variables**	**Categories**	**Frequency**	**Percentages**
		**(n)**	**(%)**
Experienced MSDs	Yes	225	84
	No	43	16
Body Area Affected	Neck	14	64
	Shoulder	11	51.6
	Upper back	71	31.6
	Lower back	141	62.7
	Wrist/Hands	95	42.2
	Elbows	17	7.6
	Hips	19	8.4
	Knees	12	5.3
Musculoskeletal pain frequence	Feet/ankles	20	8.9
	Daily	11	4.9
	Several times a week	150	66.7
	Weekly	36	16
	monthly	28	12.4
Musculoskeletal pain severity	Mild	97	43.1
	Moderate	116	51.6
	Severe	12	5.3

**Table 3:** Association between MSDs and demographic characteristics

**Variables**	**Categories**	**Experiencing MSDs**		**P-value**
		**Yes (%)**	**No (%)**	
Age In Years	20-29	59(79.7)	15(20.3)	0.114
	30-39	114(82.6)	2417.4)	
	40-49	44(95.7)	2(4.3)	
	≥50	8(15.7)	43(84.3)	
Gender	Male	166(84.3)	31 (15.7)	0.819
	Female	59(83.1)	12 (16.9)	
Years Of Practice	0-5	67 (78.8)	18 (21.2)	0.24
	10-Jun	93 (89.4)	11 (10.6)	
	15-Nov	49 (81.7)	11 (18.3)	
	16 And above	16 (84.2)	3 (15.8)	
Type of practice	Dental surgeon	60 (81.1)	14 (18.9)	0.172
	Dental therapist	149 (83.7)	29 (16.3)	
	specialist	16 (100.0)	0 (0.0)	
Geographical Area	Kigali city	129(86.5)	20(13.4)	0.139
	Out of kigali	95(73.6)	24(18.6)	
Facility Type	District hospital	63 (81.8)	14 (18.2)	0.057
	Referral hospital	33 (97.1)	1 (2.9)	
	Private clinic	91 (79.8)	23 (20.2)	
	Community health centers	6 (66.7)	3 (33.3)	
Religion	Christian	152 (86.4)	24 (13.6)	0.224
	Adventist	37 (75.5)	12 (24.5)	
	Muslim	24 (80.0)	6 (20.0)	
	Others	12 (92.3)	1 (7.7)	

**Table 4:** Association between MSDs and work-related factors

**Variables**	**Categories**	**Experiencing MSDs**		
		**Yes (%)**	**No (%)**	**P-value**
Working hours/day on dental procedures	<4 hours	7 (2.6%)	0 (0.0%)	
	4-6 hours	56 (20.9%)	17 (6.3%)	
	6-8 hours	121 (45.1%)	18 (6.7%)	
	8+ hours	41 (15.3%)	8 (3.0%)	0.16
Number of patients per day	0-5	13 (4.9%)	4 (1.5%)	
	6-10	76 (28.4%)	22 (8.2%)	
	11-15	103 (38.4%)	11 (4.1%)	
	16-20	25 (9.3%)	4 (1.5%)	
	20+	8 (3.0%)	2 (0.7%)	0.117
Number of working days/week	1-2	5 (1.9%)	5 (1.9%)	
	3-4	53 (19.8%)	7 (2.6%)	
	5-7	167 (62.3%)	31 (11.6%)	0.009
Hand scaling performance	Yes	75 (28.0%)	16 (6.0%)	
	No	150 (56.0%)	27 (10.1%)	0.623
Operator's chair adjustable	Yes	200 (74.6%)	38 (14.2%)	
	No	25 (9.3%)	5 (1.9%)	0.922
Operator's chair has arm support	Yes	109 (40.7%)	30 (11.2%)	
	No	116 (43.3%)	13 (4.9%)	0.01
Operator's chair has back support	Yes	175 (65.3%)	36 (13.4%)	
	No	50 (18.7%)	7 (2.6%)	0.383
Patient chair adjustable	Yes	187 (69.8%)	34 (12.7%)	
	No	38 (14.2%)	9 (3.4%)	0.523
Frequency of taking breaks	Every 2 hours	24 (9.0%)	13 (4.9%)	
	Every 3-4 hours	96 (35.8%)	20 (7.5%)	
	Never	105 (39.2%)	10 (3.7%)	<0.001
**Variables**	**Categories**	**Experienced MSDs**		**P value**
		**yes**	**no**	
Repetitive movement engagement	Yes	200 (74.6%)	36 (13.4%)	
	No	25 (9.3%)	7 (2.6%)	<0.001
Frequency of repetitive movements	Constantly	37 (13.8%)	3 (1.1%)	
	Frequently	134 (50.0%)	26 (9.7%)	
	Occasionally	29 (10.8%)	7 (2.6%)	0.291
Pre-existing conditions	Yes	14 (5.2%)	5(1.9%)	
	No	211 (78.7%)	38 (14.2%)	0.206
